# Global insect decline is the result of wilful political failure: A battle plan for entomology

**DOI:** 10.1002/ece3.9417

**Published:** 2022-10-12

**Authors:** Philip Donkersley, Louise Ashton, Greg P. A. Lamarre, Simon Segar

**Affiliations:** ^1^ Lancaster Environment Centre Lancaster University Lancaster UK; ^2^ School of Biological Sciences The University of Hong Kong Hong Kong SAR China; ^3^ Faculty of Science, Biology Centre of the Czech Academy of Sciences University of South Bohemia Ceske Budejovice Czech Republic; ^4^ ForestGEO Smithsonian Tropical Research Institute Ancon Panama; ^5^ Harper Adams University Newport UK

**Keywords:** cultural ecosystem services, ecosystem services, entomo‐literacy, insect decline, millennium ecosystem assessment

## Abstract

The Millennium Ecosystem Assessment assessed ecosystem change, human wellbeing and scientific evidence for sustainable use of biological systems. Despite intergovernmental acknowledgement of the problem, global ecological decline has continued, including declines in insect biodiversity, which has received much media attention in recent years. Several roadmaps to averting biological declines have failed due to various economic and political factors, and so biodiversity loss continues, driven by several interacting human pressures. Humans are innately linked with nature but tend to take it for granted. The benefits we gain from the insect world are broad, yet aversion or phobias of invertebrates are common, and stand firmly in the path of their successful conservation. Providing an integrated synthesis for policy teams, conservation NGOs, academic researchers and those interested in public engagement, this article considers: (1) The lack of progress to preserve and protect insects. (2) Examples relating to insect decline and contributions insects make to people worldwide, and consequently what we stand to lose. (3) How to engage the public, governmental organizations and researchers through “insect contributions to people” to better address insect declines. International political will has consistently acknowledged the existence of biodiversity decline, but apart from a few narrow cases of charismatic megafauna, little meaningful change has been achieved. Public values are reflected in political willpower, the progress being made across the world, changing views on insects in the public should initiate a much‐needed political sea‐change. Taking both existing activity and required future actions, we outline an entomologist's “battle plan” to enormously expand our efforts and become the champions of insect conservation that the natural world needs.

## Graphical abstract

As a subject of substantial research and media attention, the “Insect Armageddon” is both timely and somewhat controversial. Published studies argue back and forth over the extent of insect declines worldwide. Little attention has been given over what we stand to lose, should we as a species continue to ignore global insect biodiversity loss. We present a battle plan for entomologists to confront this loss and engage the world in saving insects for the benefit of all.

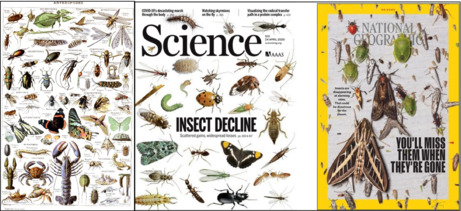

## 1 INTRODUCTION: INTERGOVERNMENTAL (IN)‐ACTION

The past 30 years of international politics have produced at least 32 reports, reviews and treaties looking to implement biodiversity targets (Buchanan et al., 2020; Johnson et al., 2017; Xu et al., 2021). Frustratingly, it is neither novel nor surprising to state that none of these efforts has actually resulted in reverse biodiversity loss trends or meaningful change in how we are exploiting the planet.

Intergovernmental institutions like the UN's Convention on Biological Diversity, the Strategic Plan for Biodiversity 2011–2020, The Millennium Ecosystem Assessment (MEA) (2005) and the 2020 Aichi Targets have documented insect biodiversity decline, for decades (Forister et al., 2019). As the esteemed Greta Thunberg has said, the combined efforts to respond to these declines has equivocated to “a lot of blah, blah, blah” and no effective response. There are strong economic arguments for an increased recognition that the natural world is both at risk and irreplaceable (Vazquez‐Brust & Sarkis, 2012). In this article we consider: (1) The proximate factors that have stood in the way of the necessary meaningful change. (2) Existing examples of effective responses happening “below” the intergovernmental level. (3) Some examples of less frequently discussed services invertebrates provide and how we can use these as opportunities to motivate better management in the future.

## 2 INSECTS ARE DECLINING WORLDWIDE

Global trends in biomonitoring have provided evidence that insects are declining, with reductions in abundance, diversity and biomass (Forister et al., 2019; Hallmann et al., 2017; Harvey et al., 2020; Kawahara et al., 2021; Wagner, Grames, et al., 2021). Furthermore, concomitant declines in species richness and abundance of insectivorous birds and insects has been detected in many regions across the world (Ceballos et al., 2017; Leclère et al., 2020; Rosenberg et al., 2019).

Critically, these conclusions come from robust datasets (Kunin, 2019), spanning decades of monitoring effort (Hallmann et al., 2020; Macgregor et al., 2019; Roth et al., 2020; Skarbek et al., 2021), and global assessments of taxa‐specific datasets (Balfour et al., 2018; Hallmann et al., 2017; Zattara & Aizen, 2021). As Forister et al. (2019) succinctly put it: we know enough about insect declines to act now.

Arguably, the widespread public awareness of insect abundance and biodiversity decline was a result of media coverage of the Sánchez‐Bayo and Wyckhuys (2019) meta‐analysis, and it would be disingenous to not mention the controversies surrounding this focal study. Despite the number and breadth of studies clearly demonstrating a global trend in insect biodiversity loss, the Sánchez‐Bayo and Wyckhuys (2019) study remains a crucible for public and political understanding of these trends, almost certainly due to its “sensationalist” and “headline grabbing” findings (Komonen et al., 2019; Montgomery et al., 2020; Thomas et al., 2019).

The combination of widespread media misinterpretation of the results of the study (Didham et al., 2020; Saunders et al., 2020), statistical errors (Daskalova et al., 2021) and methodological issues in the meta‐analysis (Haddaway et al., 2020; Mupepele et al., 2019) have been highlighted in responses to this study. Yet, each of these studies do not detract from a widespread scientific consensus on global biodiversity decline in invertebrates, though understanding the scale and trajectory of such declines is critical in mounting an appropriate level of response (Cardoso et al., 2019; Didham et al., 2020).

## 3 WHY ARE DECLINES HAPPENING?

The plethora of factors threatening insect abundance and species richness are largely well understood and have been extensively documented: land‐use change (especially habitat destruction), climate change, deforestation, habitat degradation, invasive species, urbanization and pollution to name but a few (Hallmann et al., 2020; Wagner, Grames, et al., 2021; Warren et al., 2021). Ultimately these drivers largely result from economic overexploitation. Locally and regionally, insects are challenged by additive stressors, such as insecticides, herbicides, urbanization, and light pollution (Vanbergen et al., 2013; Wagner, 2020; Wagner, Fox, et al., 2021). Drivers such as climate change, may benefit some insect species by increasing the area of suitable habitat, while being detrimental to others (Pina & Hochkirch, 2017; Pyšek et al., 2020; Schowalter et al., 2021). Socioeconomic factors that drive inaction (or in some cases active pursuit) toward mitigating climate change are nothing new (Matthews et al., 2015; Nerlich, 2010). Overall, results highlight a universal trend toward “homogenization” of biodiversity (Clavel et al., 2011; Piano et al., 2020), with climatic generalist species outcompeting specialists, leading to seemingly stable numbers that could be hiding a loss of the “little things that run the world” (Wilson, 1987). The modern “food system” sits within a global agricultural economy directed toward ever‐increasing yields to feed a growing human population (Godfray et al., 2010; Willett et al., 2019). Agricultural intensification: massive scale monoculture, fertilizer overuse, pesticide (herbicide, insecticide and fungicide in particular) and destruction of native habitats for insects in and around farmland are each pursued with the intention to increase productivity (Tilman et al., 2011). In environmental terms, the effects of pesticides and other agrichemicals are not restricted to target systems, with run‐off from agricultural systems causing widespread issues in aquatic habitats (Schulz & Liess, 1999) and Ivermectin presence in dung impacting on dung insect communities (Wall & Strong, 1987). Nor are their effects restricted solely to the direct impacts of these pesticides, indeed many of the other destructive practices of agricultural intensification act synergistically with pesticide applications (Dance et al., 2017; González‐Varo et al., 2013; Habel et al., 2019). Despite our ever increasing knowledge around historic failures to account for the unintended consequences of chemical pesticides (Sgolastra et al., 2020), no effective change to risk assessment policy has emerged following the neonicotinoid crisis. Though some may argue there are reasons to be optimistic, as wide‐ranging new plans emerge to reduce pesticide use (Kegley et al., 2010), the reality of ever‐increasing land area being exposed to chemical pesticides (Fera, 2021) and application volumes (Roser, 2021) shows us reality has not lived up to these hopes ‐ a common theme when economic arguments meet environmental reality (Forsyth, 2018; Godfray et al., 2010; Petrescu‐Mag et al., 2019; Scoones, 2016).

These complex interdependent factors also exist within a context of wilful choices by governments, industries and society: biodiversity declines may be considered unintended consequences. Human wellbeing will likely always trump nature conservation, until we pass such a point that flatlining ecosystems are detrimental to our species. Ensuring that forests are protected and that coal stays in the ground requires two ingredients that should be pervasive: direct incentives to prevent land use change in the tropics and forward thinking at a societal level. Intergovernmental action displays a certain inertia, kicking in only when change becomes impossible to ignore, this is perhaps a by‐product of a current economic approach that ignores the irreplaceability of nature (see figure 1 in Costanza et al., 1997). Linking human wellbeing with nature conservation, particularly that of insects, first requires us to come to terms with the societal perception of insects.

## 4 ENTOMO‐BIAS: DETRIMENTAL IMPACT OF SOCIETAL PERCEPTION OF INSECTS

There is no “one size fits all” strategy for engaging the public and politicians with the decline of insects. Increasing society's knowledge about insects is beneficial for convincing people that insects are more than just “creepy crawlies”, and to comprehend, intellectually, the tremendous importance of preserving insect populations (Basset & Lamarre, 2019). Public engagement with insect decline and conservation approaches necessarily requires a normalizing insect‐driven curiosity. Five key engagement aims are consistently identified across global systems that must be overcome in order to achieve an “entomo‐literate” society (Weaver et al., 2017). These are public engagement and understanding of:

1. The role of insects in the functioning of ecosystems and the recognition of insects’ benefits to humanity.
2. The dominance of insects in global biodiversity and the need for more awareness for nature conservation.
3. The current and the future environmental collapse cascades due to the interdependency of insect‐associated food webs.
4. The importance of increasing public empathy toward insects through biological education and associated outdoor and indoor activities.
5. The potential to replace traditional sources of animal proteins with more insect‐based diets, eating insects could make people value them more.

Arguably, the widespread perception of insects as “creepy crawlies” is reflected in political organizations, given the lack of coherent policy for insects on the part of governments worldwide (beyond policies for bees—notably, not all pollinating insects). There is an important role for entomologists to reach out and connect through education programs at schools and in wider society.

Narrower endeavors are emerging across the world, growing in popularity alongside “no‐mow” road verges (Phillips et al., 2020), public pollinator and butterfly gardens are becoming widespread and have been highlighted as an important insect‐conservation resource (Vickery, 1995). Gardens cannot replace natural habitat for butterfly species, since they cannot provide all larval plants necessary for the survival of various species (Di Mauro et al., 2007) and based on UK studies, the “rarest and most endangered kinds of … butterflies just do not visit gardens” (Vickery, 1995). Importantly, gardens serve as meaningful resources for people to experience the natural world and can be used to engage people in citizen science programs for biodiversity monitoring (Dennis et al., 2017; Pendl et al., 2021). This is further complimented by the growing social media followings of insect photography, species identification and naturalist groups (Basset & Lamarre, 2019; Saunders et al., 2020). Finally, insect husbandry and pet ownership has long been an enormous enterprise in Japan, with an increasing uptake worldwide (Markee et al., 2021). Though governments have failed insects, there is possibly a “silver lining” to ground‐up interest in insects driving societal change.

**FIGURE 1:**
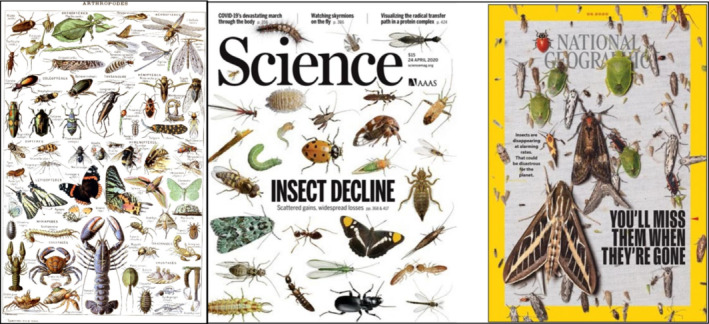
Recent articles in Science (April 2020) and National Geographic (May 2020) addressing concerns over declining insects (right, extracted from a publication from Smithsonian ForestGEO Arthropod Initiative) and an illustration (left) of some arthropoda by entomologist Adolphe Philippe Millot (1857–1921), from the Muséum National d'Histoire Naturelle of Paris. Has our perception toward insect changed from now (2020) and the time when Adolphe released this painting in the early 1900s?

Entomology is a vast and old field, yet one that has never been more relevant, and neither has its teaching (Figure 1). Taxonomic bias in ecological research is not a recent phenomenon (Leather, 2009), yet it is important to highlight that insufficient funding for entomological science has severely limited development of our knowledge of this most diverse group of terrestrial macro‐organisms (Rosenthal et al., 2017; Troudet et al., 2017). Addressing academic bias first may help with creation of a collegiate united front in support efforts to reverse insect biodiversity decline, yet making this an exclusive focus risks creating a policy “echo chamber”. Only through combining academic and public interests can effective change be achieved. Fortunately, these sectors overlap in natural history museums.

Globally, entomological collections in natural history museums are critically underfunded and currently offer an impoverished example of the natural world, despite their crucial importance to both researchers and public engagement (Salvador & Cunha, 2020). Recent advances in “Museum genomics” highlight the centrality of insect collections in understanding the past (Mayer et al., 2021). Despite the ongoing efforts of conservationists, a truly impactful strategy to engage governments and the public with saving insects has thus far failed, or had limited success (Heinemann & Weiss, 2018). Immediate and sustained governmental support of natural history museums at national and regional scales should be a policy priority. The “end goal” of such a drive would be to create an “entomo‐literate” society worldwide. The question remains: do we start with leaders or the people?

## 5 INSECTS CONTRIBUTION TO PEOPLE: WHAT DO WE STAND TO LOSE?

The potential for global declines in insect diversity and abundance is deeply concerning for reasons apparent to any entomologist or ecologist. However, to fully appreciate the case for better policy approaches, public engagement and conservation of insects worldwide, the roles of insects in “nature's contributions to people” (Díaz et al., 2018) can be divided into: (i) ecosystems; (ii) human diets; (iii) cultural and societal contributions; and (iv) the “unknown.”

### 5.1 Insect contribution to ecosystem services

Insects underpin central biotic interactions in terrestrial ecosystems. Roughly, 75% (Price, 2002) of terrestrial macro‐biodiversity is supported by a complex system of interactions between plants (hosts), herbivorous insects and their associated predators and parasitoids (often other arthropods). They also compose the bulk of the food sources for birds, reptiles, fishes and many other vertebrates (DeAngelis, 1992). Insects and other arthropods deliver numerous beneficial services in the functioning and the maintenance of our natural and anthropogenic habitats, which have been extensively documented by specific reviews (Morimoto, 2020; Ollerton, 2017; Prather & Laws, 2018; Reilly et al., 2020; Tscharntke et al., 2002).

### 5.2 Insect contribution to human diets

When taking a global perspective, edible insects have existed for millennia. The UN and European Union have officially endorsed insects as a protein source for human consumption (Mlcek et al., 2014). Uptake in more cultures (primarily European) presents a conservation momentum opportunity whereby we as entomologists can point to insects as a unique solution to a critical problem with the global food system.

Conventional livestock farming is considered to be one of the major causes of biodiversity loss worldwide (Tscharntke et al., 2012). It is extremely expensive in terms of water, surface area, energy (transport, processing, etc.) and plant resources (food). Conventional dairy and cattle agriculture requires 8 kg of feed to produce only 1 kg of body biomass (Godfray, 2011). Insects have high food conversion efficiency; on average, insects can convert two kilograms of food into 1 kg of insect mass, ready to be transformed into protein flour (FAO, 2009). They feed on bio‐waste, including waste food and human waste, compost and animal slurry, resources available for bioconversion and produced continuously by our societies (Fahrenkamp‐Uppenbrink, 2016; van Huis et al., 2015).

Insects represent a high‐quality source of protein and nutrients comparable to meat and fish (Van Huis, 2016). They are already consumed worldwide and form an important source of fiber and minerals such as copper, iron, magnesium, manganese, phosphorous, zinc and calcium that they contain (Rumpold & Schlüter, 2013). Insects can also serve as a food source for livestock feed. They represent a more sustainable alternative that has many advantages when it comes to reducing the overall impact of agriculture on the environment (Van Huis, 2016).

Insects were an important part of the diet in pre‐agriculture hunter‐gatherer societies in Africa, Asia and the Americas. DeFoliart (1999) theorizes that insects were less competitive as food items in developing areas of the Western world because the spread of agricultural practices made other sources of protein easier and more efficient to harvest. Entomophagy companies have flourished in countries where insects have been traditionally consumed. The industry is gaining momentum elsewhere, and represents a rare opportunity where consumer‐economics can align with conservation values. More insect‐derived products are now becoming available to the public (Halloran & Münke, 2014), offering a direct line to engaging the public with the value of insects to human wellbeing.

Insects have long served as traditional foods in most non‐European cultures. For example: Mopane worms important in southern Africa, Chapalines in Mexico, palm grubs eaten across tropical regions of South America, not to forget the many diverse insects eaten across southeast Asia and China (Hurd et al., 2019; Kelemu et al., 2015; Raheem et al., 2019). The question remains why the Western world has neglected insects as food and feed for so long (Morimoto, 2020). Food preferences are the result of cultural conditioning (Harris & Ross, 2009). Market models revealed that Western consumers' acceptance of edible insects is the main barrier for edible insect commercialization (DeFoliart, 1999). Without falling into a Euro‐centric trap, we must acknowledge trends around Western attitudes: acculturation toward Western lifestyles tends to cause a reduction in the use of insects. Correspondingly however, efforts by entomologists to acclimatize Western cultural perception in favor of entomophagy may provide an opportunity to engage with entomo‐literacy across environmental, conservation and sustainability platforms. The European Commission is aware of this trend, and has consequently introduced legislation in 2021 legitimizing the use of insect meal as a food source within this economic market space. Entomologists worldwide should consider the publication of this legislation as a “call to arms” for advocating insect conservation through this emergent trend in novel sustainable food production.

### 5.3 Insect contributions to wellbeing and culture

Within the scientific literature, we are now beginning to engage in meaningful discussion about the psychological, social and emotional value of viewing nature (Honold et al., 2014). Closer contact with nature can be facilitated through living in environments with a high percentage of green space (Maas et al., 2006) or through having access to nearby green areas and parks (Cohen‐Cline et al., 2015). Public green areas (city parks, nature reserves, areas of outstanding natural beauty), private green areas (domestic gardens) and smaller elements such as street trees all aid in stress reduction (de Vries et al., 2003) through facilitating connection to nature (Nielsen & Hansen, 2007; Taylor et al., 2015). Though relatively understudied, the contributions by insects to our sociological, psychological and cultural wellbeing nevertheless cannot be understated. There is some limited quantitative data on relative perception of insects with respect to other urban wildlife: interestingly, placing butterflies above hedgehogs, badgers and bats in terms of relative appeal (Bjerke & Østdahl, 2004).

The link between insects and mental wellbeing has yet to be comprehensively explored within the academic literature; though there have been suggestions to use the psychological benefits of seeing nature (specifically insects) as an effective means to engage with the public (Perrin & Benassi, 2009). One can argue that insects are important because some people like to see them and therefore get tangible benefits and a mental health boost from them. Or alternatively, their keystone role in ecosystems and green spaces may translate to a keystone role in creating the natural environments that are so beneficial. After all, reciprocal adaptation between insects and plants has driven the evolutionary trajectory of many traits generally seen as highly beneficial to humans (e.g. phytochemical diversity, attractive flowers and edible fruits).

The issues of habitat quality and biodiversity as an end goal of conservation programs have long been acknowledged in the ecological literature; indeed insects have often been championed as key indicators of environmental decline or restoration (Derhé et al., 2016; Filgueiras et al., 2015). Green space quality is equally important within public health research, though it is less a secondary consideration over quantity (Carrus et al., 2015; Honold et al., 2014). At the time of writing, however, there is a distinct paucity of studies that consider the combined contribution of insects to high‐quality habitat and the “knock‐on” effects they have on human mental health. Though conservation bodies such as the Royal Society for the Protection of Birds (RSPB) espouse the importance of insects for maintaining wildlife in the garden, relatively little attention is given to the psychological benefits of visiting butterflies, pollinating bumblebees or the glow of fireflies in the summer. These contributions have seen no lack of acknowledgement in English literature and inclusion in Japanese Haiku. 18th century Tokyo‐based poet Kobayashi Issa produced many haiku on insects. John Keats, Emily Dickinson and William Blake are just some esteemed poets that cover diverse orders of insects (Orthoptera, Diptera and Coleoptera, respectively). Poems such as Andrew Marvell's “The Mower to the Glow‐Worms”, reflecting someone turned away by humanity finding solace in the natural world. Cultural values are an integral part of Ecosystem Services and Natures Contributions to People (Ellis et al., 2019; Huntsinger & Oviedo, 2014; Pascual et al., 2017; Plieninger et al., 2015). From poetic works like these, and continued work within the humanities around insects (music, artwork, literature, videogames), insects play a significant role in shaping cultures worldwide. Conservation, ecological and entomological societies can more effectively use this sector by offering support to artists and commissioning works to celebrate those which focus on the insect world.

### 5.4 The “unknowable” contributions of insects

The “unknown knowledge” from insects (even common species) still to be discovered that represents the greatest loss if declines continue (Stropp et al., 2020). What do we potentially miss out on as a result of increased rates of extinction?

Possibly one of the most high‐profile discoveries from the insect world would be the concept of “Bee Venom Therapy”, using the peptides produced for the honeybee stings (melittin, apamin, adolapin) in treatments for various conditions including arthritis, rheumatism and even cancerous tumors (Son et al., 2007). This pharmacological discovery provides a model for other potential medical treatments derived from insects in the future, and most importantly one that is directly appealing to industry.

Insects have also served as innovative models in the fields of biomimetics, cosmetics, textiles, optical communication, imaging and nanotechnology, such as micro‐color reflectors developed using principles derived from Morpho butterfly wings (Chung et al., 2012). Scientists have also discovered that one well‐adapted Tenebrionid beetle of the dry Namib Desert, can harvest water from the air. The principle consists of a combination of hydrophilic (water attracting) and hydrophobic (water repelling) areas present on the forewings of the beetle (e.g., micro‐sized grooves or bumps). These distinct structures have evolved to increase fog and dew‐harvesting efficiency in the driest part of the world (Kostal et al., 2018). Among the 1.5 million estimated species of beetles, this is the only one that has so far provided a working model to inform the development of synthetic surfaces to harvest water.

Biological knowledge of model insect species has been exploited by medical research in molecular and cellular biology, such as in the study of the immune system (Hoffman & Brydges, 2011). After the discovery of the activation of innate immunity, this research was awarded the Nobel Prize for Medicine (Volchenkov et al., 2012). Insects are also model organisms in forensic sciences (Krinsky, 2018) and medical research (Srivastava et al., 2009). Knowledge relating to insect food‐webs has also underpinned biocontrol, with many commercially successful agents having been identified from field populations. It is extremely likely that many more are out there (DeFelice, 2004).

Overall, numerous models are yet to be discovered (chemical derivatives of insect toxins, behavioral models, thermoregulation and cryobiology for example). Conservative estimates have suggested we have described only one‐fifth of insect diversity on the planet (Basset et al., 2012). The “unknowable” contributions of insects may also be “unquantifiable”. Scientists may find that these unknown assets are difficult to highlight to the public in earnest, given the inherent lack of data on them. Yet, their mystery and allure can be used effectively by science communicators to raise interest and excitement.

## 6 INSECT CONSERVATION: WHAT CAN WE DO WITH WHAT WE HAVE?

Successful programs that have been undertaken required great efforts to accommodate ecological, climatic, geographic, cultural and political differences in insect conservation and education around the globe. These resources exist as powerful examples for demonstrating the non‐ecosystem service benefits of insects, the consequences of ongoing ecological collapse, as well as educational resources for engaging the public in insects as “beautiful, friendly creatures” and not “creepy crawlies”. They also act as important lessons for future works engaging the world with insect conservation.

A pioneering example of targeted insect conservation are the efforts to protect some species of wētā (Orthopteroids in the families Rhaphidophoridae and Anostostomatidae). Wētā are part of New Zealand's iconic endemic fauna and include some of the heaviest insects in the world. There are around 80 species including cave wētā, giant wētā, tree wētā and tusked wētā which inhabit a range of ecological niches (Gibbs, 1998). 16 species are classified as of conservation concern on the ICUN red list (Gibbs, 1998).

**FIGURE 2:**
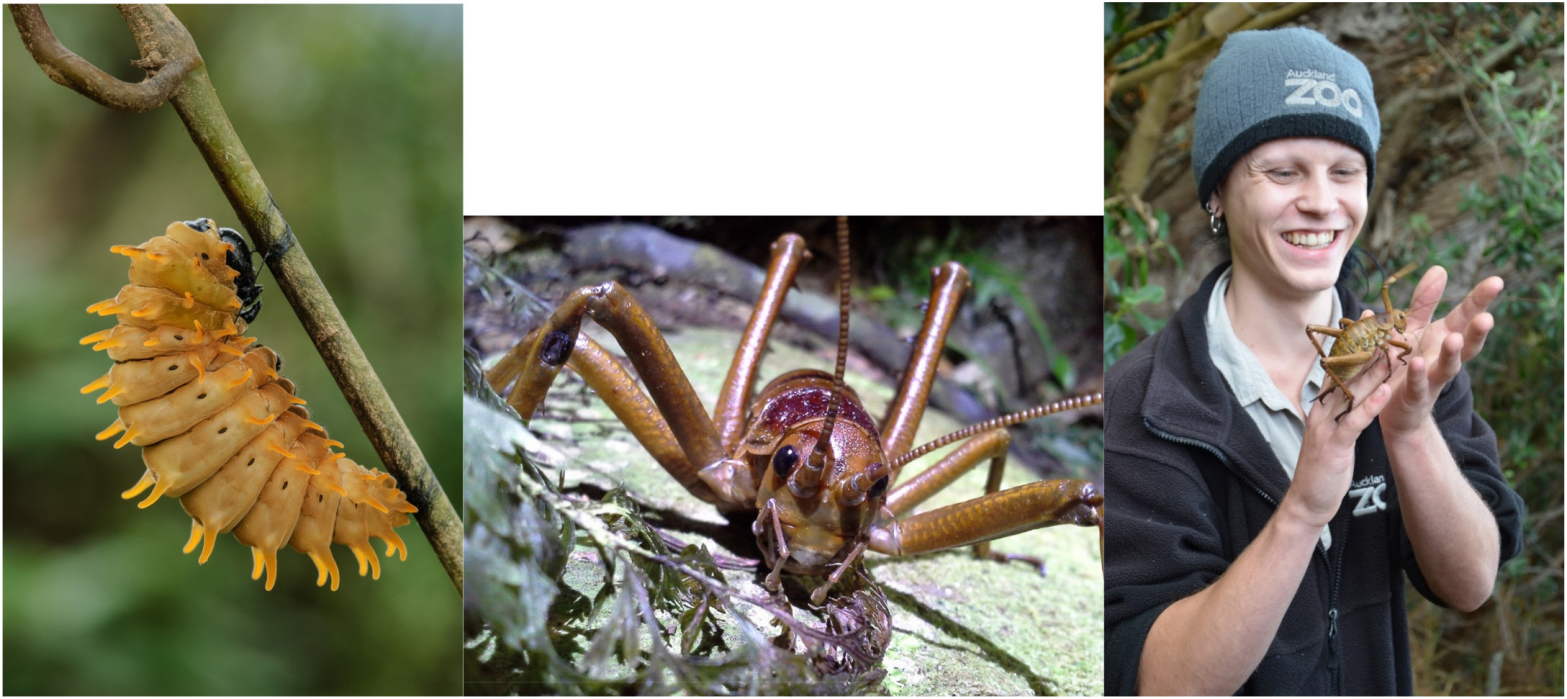
Larvae of the Kinabalu birdwing butterfly (*Troides andromache*) and Little Barrier Island giant wētā (*Deinacrida heteracantha*). Conservation actions for these species include increasing food plants, ecotourism and development of breeding programs (photo: Stephen Sutton).

The arboreal wētāpunga (or Little Barrier Island giant wētā, *Deinacrida heteracantha*), the largest species of wētā, until recently was restricted only to Te Hauturu‐o‐Toi (Little Barrier Island) (Gibbs & McIntyre, 1997). In 2018, the New Zealand Department of Conservation initiated a program to breed wētāpunga, to create self‐sustaining populations on predator‐free islands. This successful breeding program based at Auckland Zoo and Butterfly Creek, is ongoing (Figure 2), with continued translocations to predator‐free islands. Similar to larger mammal species reintroductions and translocations, this offers a case study for insect species reintroductions in tropical environments.

Some wētā species have been readily bred in zoos, however, genetic bottlenecks from small captive populations are a potential issue (White et al., 2017). In addition to breeding and release, conservation efforts have also focused on increasing wētā habitat such as the Mahoeuni Giant Wētā Scientific Reserve (Watts et al., 2011; Watts & Thornburrow, 2009). As with many conservation efforts, short‐term funding cycles can limit the long‐term sustainability of wētā conservation projects (Sherley, 1998). These are not uncommon issues with animal conservation, but do still present key lessons that issues with genetic bottlenecking and short‐term funding extend to invertebrate conservation.

Other examples of insect captive breeding and reintroduction include a program to prevent the extinction of the world's largest butterfly—Queen Alexandra's Birdwing (*Ornithoptera alexandrae*). This species is endemic to the Popondetta region of south‐eastern Papua New Guinea and is classified as endangered on the ICUN red list, primarily as a result of habitat loss (Parsons, 1992). New Britain Oil Palm Limited (NBOPL), a member of the Roundtable on Sustainable Palm Oil (RSPO) has established a breeding center in order to increase butterfly populations. This project highlights the potential for funding from industry to support insect conservation efforts. Efforts to increase host plant availably of endangered insect species have also shown some success. The Swallowtail and Birdwing Butterfly Trust (SBBT) are working to increase numbers of the Kinabalu birdwing butterfly (*Troides andromache*) in Sabah, Malaysia, by training village homestay operators to propagate host plants of *T. andromache* to attract ecotourists (Figure 2). This combination of citizen science approaches with a landscape recovery plan, focusing on environmental restoration rather than just on species relocation, can provide an example project structure for other insect conservation programs.

Another alternative model for insect conservation can be found in the Area de Conservación Guanacaste (ACG), in Costa Rica (Janzen & Hallwachs, 2009). Since 1971, Daniel Janzen's team have created a National Park in 1260 km^2^ of dry, cloud and rain forests, with the focus being on conservation of caterpillars and their parasites, rather than the typical story of a charismatic megafauna (Janzen & Hallwachs, 2021). With decades of bioinventory records, this park offers both a powerful conservation resource and a potentially effective story in presenting the plight of declining insect biodiversity in the tropics to the world (Janzen & Hallwachs, 2021).

The Wanang Conservation Area (WCA) in Papua New Guinea was awarded the United Nations Development Programme's Equator Prize in 2015. The origins of WCA can be traced back to 2000 and its conception, design and approach can be credited to local communities led by Philip Damen. The 10 local clans have secured 10,770 ha of primary rainforest against encroaching logging. This incredible achievement has facilitated the establishment of a Smithsonian Institution ForestGEO 50‐ha dynamic forest plot alongside a permanent forest research station. The overriding impression of the WCA is that of a community with a unified belief, conservation is driven by a recognition that the forest, as an entity, is worth more intact than it can ever be worth commercially (Novotny & Toko, 2015). The benefits for wildlife conservation are clear and the WCA is a model of how researchers, local landowners and NGO's can work alongside each other to advance not just entomological research, but whole forest‐scale conservation where it is most needed. Papua New Guinea's customary land rights can slow forest decline as logging concessions need to get clan‐level stakeholders on board. This highlights the need to consider carefully what local and national level laws prevail.

Local initiatives may help the public to understand the roles of insects in the functioning of ecosystems and to understand the current threats insects are facing. Many initiatives utilize common insects that are easy to observe and collect in their natural habitat, such as the British Bioblitz program (https://www.bnhc.org.uk/bioblitz/) and National Insect Week (https://www.nationalinsectweek.co.uk/). Other insect field‐based programs, such as “Des Insectes et des Hommes” (Lamarre et al., 2018) conducted in Eastern Africa, have confirmed that participants often gained a better understanding of the services provided by insects when observing them in the field (Borsos et al., 2018). Using simple taxonomic identification guides, illustrated plates and assistance from teachers, an ordinal‐level identification of insects can be a realistic objective to reach (Lamarre et al., 2018). Access to an open laboratory also enables participants to better understand insect functional traits and to envision the challenges insects face (Basset & Lamarre, 2019; Morimoto, 2020). This translation also allows us to impart greater understanding of the diverse roles that insects can play in that ecosystem. Any of these case studies of successful insect conservation, combined with lessons learned in their limitations, provides any future efforts the opportunity to build on this work previously performed. In many of these cases, the project leads are notoriously friendly and engaging individuals, highly supportive of new and emergent causes.

## 7 CHANGE ON THE HORIZON OR THE SAME MISTAKES?

Political change may be mounting, though debatably the impact on the ground to insect conservation will be non‐existent, although insects were recognized at COP26 (UKRI, 2021). Within the UK, the Environmental Land Management (ELM) scheme seeks to deliver on the targets on the 25‐year environment plan (Defra, 2020) by completely replacing schemes currently available under the EU's Common Agricultural Policy (CAP). Valuing insects based on their financial contribution to human wellbeing has led to the troublesome lack of political engagement in insect conservation. The instability in UK environment and agriculture strategies is likely to continue (Downing & Coe, 2018; Hodge, 2019). Entomologists can take the lead in advocating for more effective change in perspective and direction in policy at this critical time.

## 8 AN ENTOMOLOGIST'S BATTLE PLAN

Insect declines have attracted a great deal of public and political attention (Daskalova et al., 2021; Komonen et al., 2019; Montgomery et al., 2020). Immediate and substantial actions are needed to protect insect species in order to maintain global ecosystem stability (Eggleton, 2020). The decline of insects has drawn an unprecedented amount of media attention, driving an increased awareness of their ecological importance; along with an emotional anxiety among the public for their decline and extinction (Rowlatt, 2020). We have seen the fossil fuel industry abuse public confidence in research due to exaggerated or poorly conducted studies (Brulle, 2014). The first point in our entomological battle plan is to proactively and publicly address government inaction. Leadership from entomologists can overcome policy “turbity” today, limit the need for widespread public action akin to the “Just Stop Oil” and “Insulate Britain” disobedience campaigns seen in response to the destructive fossil fuel industry.

Many societies have become increasingly disconnected from nature, both emotionally and intellectually (Bratman et al., 2019; Caillon et al., 2017; Ives et al., 2018). We have made technological advances and solved complex problems in fields such as genetics, robotics, engineering, artificial intelligence, aerodynamic, physics, medicine and computer‐related technologies. The second point in our action plan is for advocacy in the “unknown knowledge” from insects (Stropp et al., 2020), showing what technological developments we owe to the insects, and the amount left to be discovered to bridge this gap between emotional and intellectual connection to nature.

Insect declines are on par with the rates of bird declines, plant declines, though possibly slower than mammal decline (Nichols et al., 2009; Pereira et al., 2010). The major challenges facing biodiversity loss are overpopulation, overconsumption and climate change (Díaz et al., 2018). The politics are complicated: there is considerable political will to place people, businesses, economy, status of living and social justice matters in front of those of nature's needs (Eden, 2016; Kati et al., 2015). This is linked inextricably with ongoing biodiversity loss: interventions that solely target damaging human activities without making efforts to restore their environment (Lombardi et al., 2017; Wood & Goulson, 2017) The third point in the battle plan is to present a united front, aligning insect conservation with wider efforts by bird, plant and mammal conservationists to show clear interdependencies between ecological fields and consequential benefits.

The potential impacts of long‐standing perception of insects as threatening “creepy crawlies” remains convincing, and highlights a potential route to effective societal change at all levels. The fourth point in the battle plan is to counter this perception through building on extant platforms for engaging school students with the wonders of the insect world. Outdoor activities, for instance observing and inspecting live insects, in the middle of the forest (Lamarre et al., 2018) can create a unique and memorable experience of nature (Borsos et al., 2018).

Insect conservation efforts are primarily focused on large, charismatic insect species which are of known conservation concern; however, many unknown species or entire assemblages of insects may be silently disappearing. Arguably, the last widespread and impactful “catchphrase” advocating for insects was 35 years ago when E.O. Wilson coined the phrase “The Little Things That Run the world” (Wilson, 1987). Although this had a high‐impact depth in the academic world, it did not persist in the public zeitgeist. The “Insect Apocalypse” had a sudden and widespread impact in the public zeitgeist (Simmons et al., 2019), but three years on, the world has seemingly moved on. The final point for the entomologist's battle plan is to develop the perfect simple catchphrase with longevity and impact to effectively set concern for insect wellbeing globally for the next decade or more. And then to use organic cultural osmosis and direct government lobbying across the world to ensure such a phrase achieves this potential. We as entomologists owe it to our field and to our organisms to be driving this change in the online (Leather, 2015) and offline worlds (Borsos et al., 2018).

## AUTHOR CONTRIBUTIONS

**Philip Donkersley:** Conceptualization (equal); funding acquisition (equal); project administration (equal); writing – original draft (equal); writing – review and editing (equal). **Louise Ashton:** Conceptualization (equal); visualization (equal); writing – original draft (equal); writing – review and editing (equal). **Greg P. A. Lamarre:** Investigation (equal); writing – original draft (equal); writing – review and editing (equal). **Simon Segar:** Investigation (equal); writing – original draft (equal); writing – review and editing (equal).

## CONFLICT OF INTEREST

The authors declare no conflict of interest.

## Data Availability

No data accompany this manuscript.
