# Corrigendum

**DOI:** 10.1002/ece3.9485

**Published:** 2022-11-05

In the recent article by Donkersley (2022), photo credits were missed in figure 2 caption.

The correct caption is shown below:

**FIGURE 2** Larvae of the Kinabalu birdwing butterfly (*Troides andromache*) and Little Barrier Island giant wētā (*Deinacrida heteracantha*). Conservation actions for these species include increasing food plants, ecotourism, and development of breeding programs (photo: Stephen Sutton) Photo credits: Chien C Lee (Independent Photographer)—Birdwing larvae & Richard Gibson (Aukland Zoo)—Weta photos.

The authors apologize for the error.

## References

[ece39485-bib-0001] Donkersley, P. , Ashton, L. , Lamarre, G. P. A. , & Segar, S. (2022). Global insect decline is the result of wilful political failure: A battle plan for entomology. Ecology and Evolution, 12, e9417. 10.1002/ece3.9417 36254301 PMC9555050

